# Effectiveness of a Novel Smartphone Health Education Intervention in Enhancing Knowledge, Attitudes, and Practices for the Prevention of Respiratory Tract Infections Among Private Hajj Pilgrims From Malaysia

**DOI:** 10.3389/fpubh.2021.594204

**Published:** 2021-07-01

**Authors:** Mohammed Dauda Goni, Nyi Nyi Naing, Habsah Hasan, Nadiah Wan-Arfah, Zakuan Zainy Deris, Wan Nor Arifin, Aisha Abubakar Baaba, Babagana Mohammed Adam, Muhammad Rafie Arshad

**Affiliations:** ^1^Department of Microbiology and Parasitology, School of Medical Sciences, Universiti Sains Malaysia, George Town, Malaysia; ^2^Faculty of Veterinary Medicine, Universiti Malaysia Kelantan, City Campus, Kota Bharu, Malaysia; ^3^Faculty of Medicine, Medical Campus, Universiti Sultan Zainal Abidin, Kuala Terengganu, Malaysia; ^4^Faculty of Health Sciences, Universiti Sultan Zainal Abidin, Kuala Terengganu, Malaysia; ^5^Unit of Biostatistics and Research Methodology, School of Medical Sciences, Universiti Sains Malaysia Health Campus, Kubang Kerian, Malaysia; ^6^Centre for Language Studies and Generic Development, Universiti Malaysia Kelantan, Kota Baharu, Malaysia; ^7^African Institute of One Health Research & Diagnostics (AIOHRD), Abuja, Nigeria; ^8^School of Computer Sciences, Universiti Sains Malaysia, George Town, Malaysia

**Keywords:** intervention, respiratory tract infection, knowledge, attitude and practice, Hajj pilgrims

## Abstract

This study was aimed to develop and evaluate the efficacy of a health education program for increasing knowledge, changing attitudes, and promoting prevention practices to reduce the incidence of RTIs among Malaysian Hajj pilgrims. A quasi-experimental study was done among 130 Hajj pilgrims attending Hajj orientation course organized by a private Hajj tour companies. Hajj pilgrims assigned to intervention group (*n* = 65) and control group (*n* = 65). Data were collected baseline and after return from Hajj using a validated questionnaire. Mixed design repeated measure ANOVA was used to analyse the effect of group, time, and group-time interaction on the dependent variables. There was a significant improvement in knowledge, attitude and practice scores in the intervention group compared to the control group. Based on the findings of the interaction of time and group, there was a significant statistical difference for post intervention for knowledge (*p* = 0.005), attitude (*p* = 0.041), and practice (*p* = 0.002). The newly-established health education intervention smartphone application was effective in improving KAP toward RTIs prevention among pilgrims.

## Introduction

Hajj pilgrimage is one of the largest annual mass gatherings which usually takes in Saudi Arabia with over 2 million pilgrims characterized by a high prevalence of respiratory illness among pilgrims from Malaysia and other parts of the world (1–3). There is a continued rise in the prevalence of RTIs despite the preventive practices among the Hajj pilgrims (4, 5). The COVID-19 pandemic that resulted in over 3,424,380 deaths with over 165 million people infected worldwide as at 11th August 2020 based on the figures from the Johns Hopkins University (6). The rapid transmission of the COVID-19 infection all over the world, the surge in confirmed cases as well as the lack of valid treatment and prophylaxis options, posed a serious threat to Hajj and other mass gatherings.

The usual practical approach of the protective measures appears inadequate to reduce it the incidence during the pilgrimage (7–9). Complementary and synergistic adherence to the various preventive practice such as proper hand hygiene, social distancing, vaccination, the use of face mask and cough etiquette must be ensured to achieve optimum protection (10). In Malaysia, a government Hajj Fund (Tabung Haji) is saddled with the responsibility of managing the Malaysian pilgrims during Hajj. They are the regulators and service provider for Hajj and Umrah for the pilgrims including the private tour companies.

Recent studies indicated a remarkable increase in health-related smartphone apps among the million users globally (11). Apps seem to be a befitting means of delivering practical and effective health education interventions (12). Various studies have reviewed the findings of the effectiveness of health-related apps targeting behavioral change interventions, such as physical activity and prevention of infections during Hajj as well as to trace pilgrims health behavior and obtain infectious disease data (13–16).

The gaps in the knowledge, including bad attitudes and poor practices regarding infection prevention and control, have been reported among healthcare workers during Hajj (17, 18). Some studies found out that the importance of bridging knowledge gaps concerning infectious disease diagnosis, treatment, prevention, and control have some negative attitudes and connect with health behavior (19). Therefore, people engage in poor practices, all of which contribute to their increased risk of infection and negative impacts on the community (20). A proper and well-planned health education intervention and the applicability of the acquired knowledge can serve as a base for improving the challenges, constraints and limitations, therefore directing resources and strategies toward the appropriate responses for the development of better health care.

The traditional Hajj orientation courses conducted by the Hajj agencies in Malaysia as well as the use posters, flyers, tv programmes to enlightened the pilgrims appears to be inadequate as can be seen in the increase in the incidence of the RTIs among Hajj and Umrah pilgrims. However, the importance of health education intervention module has not been evaluated among Hajj pilgrims. Also, there is no any health education intervention module via smartphone application that target behavior modification and improving preventive practices that are directed and specific for Hajj pilgrims regarding RTIs in Malaysia. Therefore, the objective of this study was to determine the effectiveness of a novel smartphone health education intervention module in improving knowledge, attitude, and practices of Malaysian Hajj pilgrims for the prevention of RTIs.

## Methods

### Study Design and Location

A quasi-experimental study was conducted among Hajj pilgrims attending Hajj orientation course organized by two major different private Hajj company in Kelantan, Malaysia. Private Hajj companies have relatively smaller number of pilgrims and therefore easy to manage and trace the study participants after returning from the pilgrimage. The study was conducted in Kelantan State, Malaysia. The sample in our research will represent ~20% of the total Malaysian pilgrims. Kelantan state is predominantly a Muslims state with about 96.8% of its population being Muslims and belongs to the Malay ethnic group. The sampling method used was cluster sampling, which was done in two stages. The first stage was a purposive selection of Hajj and Umrah companies as clusters. Each of the two companies were randomly allocated to intervention and control group, respectively. The intervention group received the health education intervention and the control group received a Hajj guide on regular Hajj routines developed by Mohamed et al. (21). Baseline data were collected from January 2019 to April 2019 before departure to Hajj and post-intervention immediately after return from Hajj. Hajj pilgrims aged 18 years and above who were willing to participate were included in the study. Pilgrim who cannot read and write and non-smartphone users were excluded from the study. This trial was guided by the Consolidated Standards of Reporting Trials (CONSORT) statement (22). In this type of study, a control group with comparable characteristics and functioning to the experimental group was selected. Randomization was not applied in this study. Open label was applied as it was not possible to blind the respondents, private Hajj companies management and investigators.

### Sample Size Calculation

The sample size for this study was calculated by applying the formula for estimating sample size in hypothesis testing by comparing two means as outlined by Lemeshow et al. (23) using sample size calculator by Arifin (24). This formula gave the required number (*n*) for each group. The estimated difference of mean knowledge score between intervention and control groups was selected based on standard deviation and effect size. Standard deviation value was taken from the pilot study, and 0.80 was selected as the value for effect size. The standard deviation of the mean knowledge score = 38.24 from the pilot study. The estimated relative difference of mean knowledge score between intervention and control groups (Δ) = 30%. The minimum sample size needed was 26 participants per group. An additional 20.0% was added after considering drop-out from the study which resulted in 26 + (26 × 0.2) = 33. However, cluster design was applied in the present study. To accommodate the design effect, the calculated sample size was multiplied by two for correction. Therefore, the sample size required in this study was (33 × 2) = 66 per group. The total required sample size was (66 × 2) = 132 pilgrims in both the intervention and control groups.

### Data Collection

Participants were recruited during the Hajj orientation course from the two different Hajj companies. A trained data enumerator collected data from Hajj pilgrims who were given written informed consent to participate in the research. The pilgrims were briefed about the purpose of the study by the enumerators in easy and unambiguous terms. A validated, pre-tested and self-administered questionnaire was administered at the beginning of the orientation course were given to the participants to fill (25). The questionnaire administered in this study validated, pretested and self-administered consisting of four sections. The sections A, B, C and D, covered socio-demographic variables, RTI related knowledge, attitude toward RTI prevention and practices related to RTI prevention. Section A: it consists of 23 statements which cover socio-demographic characteristics studied including age, gender, race and marital status, occupational status, level of education, how many times have you performed Hajj or Umrah in the last 5 years, vaccinations history, and presence of comorbidities and presence of RTI before departure to Hajj. Section B: It had 9 questions with “Yes” or “No” options includes questions on knowledge of RTIs based on etiology, transmission, risk groups, signs and symptoms, complications, the use of PPE and prevention practices. Section C includes questions on prevention attitudes regarding RTI based on the health belief model and had 11 questions. The responses were on a 5-point Likert scale with the options: strongly agree, agree, not sure, disagree and strongly disagree. Section D: consists of questions related to prevention practices toward RTIs and had statements on the preventive practices of pilgrims relating to RTIs prevention with “Always,” “Occasional,” and “Never” options. Results of the reliability test carried out showed Cronbach's coefficient alpha for knowledge, attitude and practice was 0.831, 0.777, and 0.729 respectively.

### Health Education Intervention

Hajj pilgrims traveling through one of the travel company and are attending the Hajj orientation courses were consecutively enrolled and assigned to receive health education via smartphone application as the intervention group. The health education intervention module on knowledge, attitude, and practice regarding RTIs prevention among Hajj pilgrims were developed as a smartphone application through a process of consultations with a panel of experts consisting of epidemiologist, microbiologist, health educationist, computer scientist, and medical statistician. The module was developed based on the health belief model (HBM). The HBM alluded the high predictive capacity if an individual will adopt practices to prevent diseases, if they see themselves as susceptible to the disease (perceived susceptibility), if they consider it would produce conceivably severe health outcomes (perceived severity), if they consider that a singular practice accessible to them would decrease the susceptibility or severity or lead to other positive outcomes (perceived benefits), and if they perceive few negative characteristics linked to the health action (perceived barriers) (26). The percentage of variance explained by the HBM should be enough to produce a significant effect. In addition, the educational module was tested among 10 pilgrims who did not participate in the study for clarity of meaning, language and the flow of contents. The module consisted of five main activities applied from the HBM (27). We used various behavioral change techniques to enhance the pilgrims' motivation to make lifestyle changes during Hajj. They were: first, “Perceived susceptibility of pilgrims toward RTIs”; pilgrims were educated on their perceptions toward the cause and risk of RTIs during Hajj. Second, “Perceived severity of RTIs” referred to the beliefs a person holds concerning the complications of RTIs; this technique informed the pilgrims about these effects and educated them. Third, “Perceived benefit for the prevention of RTIs”; pilgrims were educated on health behavior to adopt to prevent RTIs. Fourth, “Perceived barriers for the prevention of RTIs”; pilgrims were taught of the disadvantages or potential obstacles they must to overcome in attempting to improve their behaviors. Lastly, “cues to action for RTI prevention of RTIs”; we used an interactive, user-friendly smartphone application to stimulate awareness and change behavior.

The Health education intervention application (Hajj HEM) has several major components, including (1) pages for registering the users and collecting the individual's personal health data, which is the information users want to protect in the app; (2) pages demonstrating the overview of respiratory tract infection and prevention steps for contracting the infections. First of all, the user registers an account and key in some personal health information. Users can go to the application based on the menu options. Users can also go to the formative assessment section and use the interactive questions and answers to assess their understanding of the health education module. To overcome the challenges of lost in internet connection, the application was designed to function even without an internet connection once it is installed in the smartphone. The final version of the Hajj HEM application can be assessed through this URL https://play.google.com/store/apps/details?id=my.usm.hc.sms.hajjhem. The application was restricted to only pilgrims that consented to participate in the study.

Participants were encouraged to be compliant regarding the usage of the smartphone application before, during and after Hajj. The formative assessment section was included in the module for the evaluations of participants comprehension, learning needs and progress throughout the intervention. Formative assessments also help the researcher identify concepts that participants are struggling to understand, how participants comply with the application. Similarly, sections were provided regarding “Feedback from users about the content” and “Feedback from users about the app.”

### Measurement of Outcome Variables

The outcome variables for this study was the change in knowledge, attitude, and practice regarding RTI prevention. Knowledge had 29 questions and had “Yes” or “No” options. Correct answers attracted one mark while wrong answers were scored zero. A respondent could get scores within the range of 0–29 scores. Attitude had 12 questions measured on a five-point Likert scale with strongly disagree = 1, Disagree = 2, Not sure = 3, Agree = 4, and strongly agree = 5. The scores ranged from 12 to 60. Practices had 13 questions with “Always,” “Occasional,” and “Never” options. Each item was given score 2 for “always” response, 1 for “occasional” and 0 for “never” responses. A total possible maximum score on the practice domain was 26 and the minimum was 0. Higher scores imply a higher positive knowledge, attitude, and practice toward RTI prevention, while poor scores show mistrust of preventive strategies and/or negative views toward the need for preventive practices.

### Data Analysis

Data entry and analysis was performed using Statistical package for social sciences (SPSS) version 22 (IBM, 2014). Frequencies, percentage, mean (standard deviation) were analyzed using descriptive statistics. Chi-square test and Fisher exact test was used to analyse the association of categorical variables between intervention and control groups. However, independent sample *t*-test assuming equal variance was used to compare between the intervention and control groups. *P* < 0.05 were considered to be statistically significant. For the evaluation of the effectiveness of the health education intervention, mixed-design ANOVA was primarily the analytical method employed to understand if there is an interaction within-subjects effect, between-subjects effect and time (28). Partial eta squared (η2) was the measures of effect size. The strength of partial eta square (η2) was interpreted as small effect = 0.01, moderate effect = 0.06, larger effect = 0.14 (29).

### Ethical Issues

Ethical clearance to conduct the study was obtained from Universiti Sains Malaysia, Ethics Committee for Research Involving Human Subjects (ref no: USM/JEPeM/17020146). and Universiti Sultan Zainal Abidin Malaysia human research ethics committees (UniSZA/UHREC/2019/88). Informed written consent was obtained from each participant. In addition, the trial was registered with Australian New Zealand Clinical Trials Registry (ANZCTR) with registration number ACTRN12619000217101.

## Results

**Figure 1:**
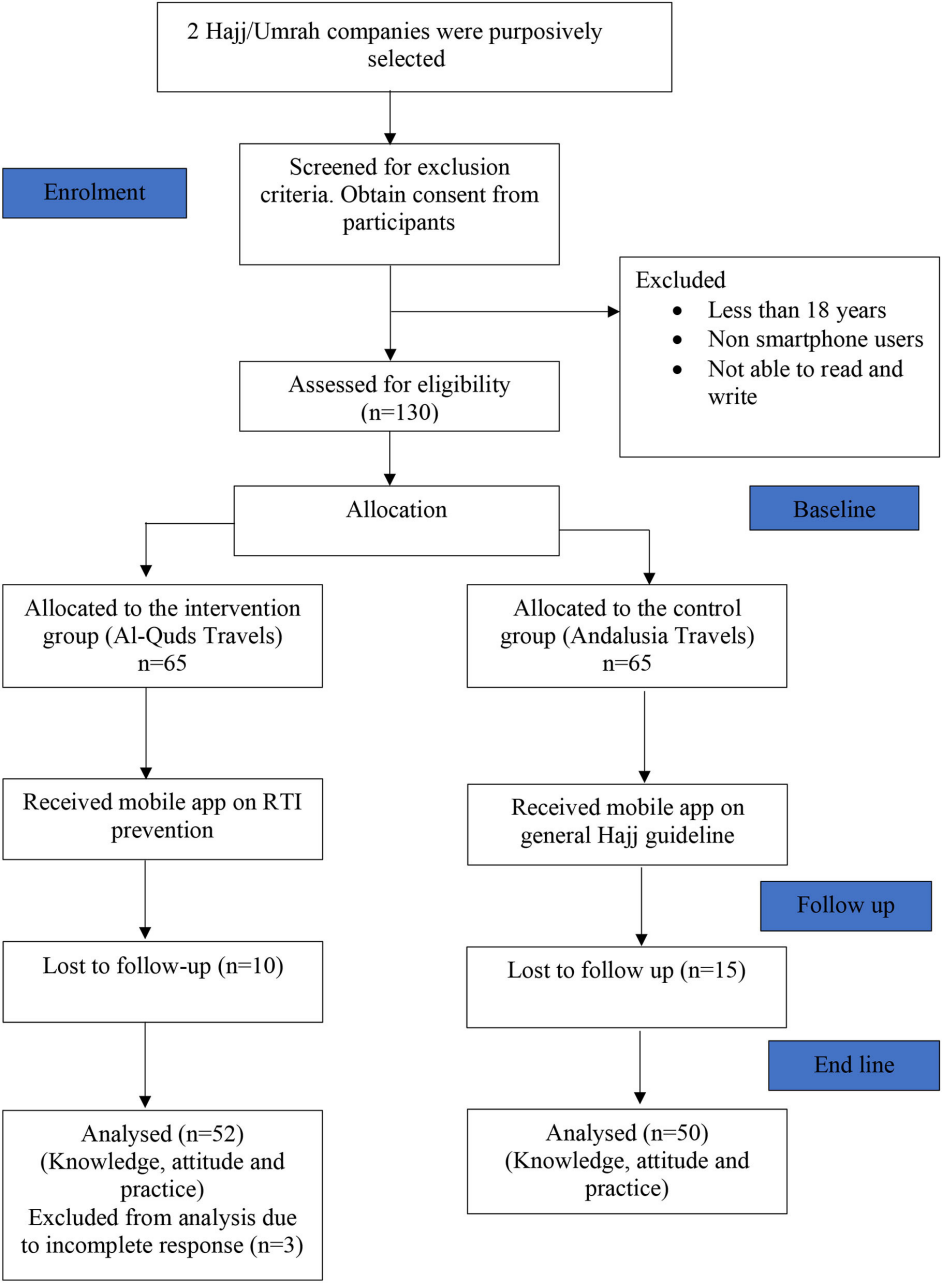
Flow chart of respondents in a quasi-experimental study conducted among Hajj pilgrims in private Hajj companies from Kelantan, Malaysia.

A total of one hundred and thirty-two (132) Hajj pilgrims were recruited for this study from 2 purposely selected private Hajj and Umrah tour companies during the pre-intervention study. These participants were grouped into intervention (65) and control (65) groups. However, during the post-intervention, 13 respondents from the intervention group and 15 respondents from control group were lost to follow up giving a response rate of 83.87 and 76.92%, respectively One of the Hajj tour company was allocated the control group, and the other tour company was allocated the intervention group to reduce contamination at baseline assessment, as shown in Figure 1.

### Socio-Demographic Characteristics by Groups

Table 1 displays the socio-demographic characteristics and the presence of RTIs symptoms of the Hajj pilgrims in the intervention and control groups at baseline. The results showed that there was no significant difference between the two groups. The control and the intervention groups were comparable at baseline. The KAP questionnaire was re-administered at three to 4 weeks after intervention upon return from Hajj. However, data was collected from 52 (80%) and 50 (76.9%) at post-intervention from both intervention and control arms, respectively. The age of the respondents during the intervention study ranged from 20 to 73 years, with a mean (SD) age of 46.90 (12.78) years. Majority of the respondents are females in both groups. Most of the respondents (76.5%) were married, while half of them (50.0%) are civil servants. In terms of the level of education, bachelor's degree, and secondary holders are the majority, and they account for 36.3% each.

**Table 1:** Socio-demographic characteristics of the respondents.

**Socio-demographic factor**	**Intervention *n* = 52**	**Control *n* = 50**	***p***
	***n* (%)**	***n* (%)**	
**Age (years)**			
Mean (SD)	48.88 (11.75)	44.84 (13.57)	0.110^a^
**Gender**			
Male	16 (30.8)	14 (28.0)	0.465^b^
Female	36 (69.2)	36 (72.0)	
**Marital status**			
Married	46 (82.7)	35 (70.0)	0.083^c^
Single	6 (11.5)	14 (28.0)	
Divorced/widowed	3 (5.8)	1 (2.0)	
**Occupation**			
Civil servant	24 (46.2)	27 (54.0)	0.692^c^
Self employed	5 (9.6)	4 (8.0)	
Private	5 (9.6)	5 (10.0)	
Pensioner	13 (25.0)	7 (14.0)	
Housewife	4 (7.7)	4 (8.0)	
Student	1 (1.9)	3 (6.0)	
**Highest level of education**			
Ph.D.	4 (7.7)	0 (0)	
Master's degree	3 (5.8)	4 (8.0)	
Bachelor's degree	16 (30.8)	21 (42.0)	
Diploma	11 (21.2)	6 (12.0)	
Secondary school	18 (34.6)	19 (38.0)	
**Previous Hajj experience**			
Yes	16 (30.8)	9 (18.0)	0.102^b^
No	36 (69.2)	41 (82.0)	
**Previous Umrah experience**			
Yes	16 (30.8)	11 (22.0)	
No	36 (69.2)	39 (78.0)	
**Influenza vaccination history**			
Yes	15 (28.8)	13 (26.0)	0.460^b^
No	37 (71.2)	37 (74.0)	
**Pneumococcal vaccination history**			
Yes	12 (23.1)	13 (26.0)	0.455^b^
No	40 (76.9)	37 (74.0)	
**Presence of influenza-like illnesses symptoms before departure**			
Yes	2 (3.8)	1 (2.0)	1.000^a^
No	50 (96.2)	49 (98.0)	
**Presence of common cold symptoms before departure**			
Yes	2 (3.8)	1 (2.0)	1.000^a^
No	50 (96.2)	49 (98.0)	

a *t-test*.

b *Fisher's exact test*.

c *Pearson Chi-square*.

### Effectiveness of Intervention on KAP Mean Score Based on Time

For the effectiveness of the health education intervention based on time effect, a significant difference of mean knowledge score in the intervention group based on time (*F* = 0.31, *p* = 0.002) was revealed in the analysis. Therefore, multiple paired *t* tests were done with adjusted α based on Bonferroni correction, as shown in Table 2. Results of the paired *t* test showed that there was a significant difference in the pre-post-test intervention (mean difference = 3.46, 95% CI: 1.29, 5.63; *p* = 0.002) for the intervention group. However, results for the control group, based on the paired *t* tests showed that there was no significant difference in the pre-post intervention (mean difference = 0.46, 95% CI: −1.22, 2.14; *p* = 0.584). In conclusion, with regards to the knowledge domain, there was a significant difference in mean knowledge score within the intervention group only based on time while the control group showed no significant difference.

**Table 2:** Comparison of KAP scores of RTI prevention during Hajj/Umrah within each group based on time (time effect).

**Comparison**	**Intervention group (*****n*** **=** **66)**		**Control group (*****n*** **=** **66)**	
	**Mean Diff (95% CI)**	***p***	**MD (95% CI)**	***p***
**Knowledge**				
Pre-post intervention	3.46 (1.29, 5.63)	0.002	0.46 (−1.22, 2.14)	0.584
**Attitude**				
Pre-post intervention	2.17 (0.16, 4.19)	0.035	0.52 (−0.99, 2.03)	0.492
**Practice**				
Pre-post intervention	2.73 (1.53, 3.93)	<0.001	0.96 (−0.29, 2.21)	0.129

*Repeated measures ANOVA within-group analysis was applied followed by pairwise comparison with 95% confidence interval adjustment by Bonferroni correction. MD, mean difference*.

In the attitude section, there was also a significant difference in mean attitude score within the intervention group based on time (*F* = 4.677, *p* < 0.001). Similarly, multiple paired *t* tests were conducted as shown in Table 2 and which indicated a significant statistical difference in pre-posttest (mean difference = 2.173, 95% CI: 0.156, 4.190; *p* = 0.035) for the intervention group only. On the other hand, for the control group, there was no significant statistical difference in the pre-post intervention. In conclusion, for attitude, there was a significant difference in the mean attitude score within the intervention group only based on time.

Whereas, for the practice section, there was also a significant difference in mean practice score within the intervention group only based on time (*F* = 20.989, *p* < 0.001). Multiple paired *t* tests were performed (Table 2) and showed that there was a significant difference in the pre-posttest (mean difference = 2.73, 95% CI: 1.53, 3.93; *p* < 0.001) of the intervention group. While for the control group, paired *t* tests showed that there were no significant differences in the pre-post (mean difference = 0.96; 95% CI: −0.29, 2.21; *p* = 0.129). In conclusion, there was a significant difference in the mean practice score within the intervention group only based on time.

### Effectiveness of Intervention on KAP Mean Scores Based on Group Regardless of Time

From Tests of Between-Subjects Effects, the findings of this study indicated there were no any significant differences of mean knowledge, attitude, and practice scores between intervention and control groups (*p* = 0.169, *p* = 0.101, and *p* = 0.078, respectively) regardless of time. The mean knowledge, attitude and practice scores were higher in the intervention group compared to the control group (Table 3). However, the results also showed the main effect for the between-group factor analyses (partial η2) based on knowledge, attitude and practices scores as 0.019, 0.027, and 0.031 respectively based on the cut-off points for 0.01 (small), 0.09 (medium), and 0.25 (large) (30).

**Table 3:** Comparison of KAP scores of RTI prevention among Hajj/Umrah pilgrims between groups (Group effect regardless of time).

**Comparison**	**Mean (95% CI)**	**F-stat (df)**	***p***	**Partial Eta Squared**
**Knowledge**				
Control	16.15 (14.81, 17.49)	1.92 (1)	0.169	0.019
Intervention	17.46 (16.15, 18.78)			
**Attitude**				
Control	31.96 (30.77, 33.16)	2.74 (1)	0.101	0.027
Intervention	33.36 (32.18, 34.53)			
**Practice**				
Control	26.58 (25.38, 27.78)	3.18 (1)	0.078	0.031
Intervention	28.10 (26.92, 29.28)			

*Repeated measures ANOVA between-group analyses were applied Level of significance was set at 0.05 (two-tailed)*.

### Effectiveness of Intervention on Mean KAP Scores Based on Time-Group Interaction (Group Effect With Regard to Time)

Based on the findings of the interaction of time and group, there was a significant statistical difference for post intervention based on estimated marginal means for knowledge (*p* = 0.005), attitude (*p* = 0.041), and practice (*p* = 0.002) as shown in Table 4. However, for pre intervention, the estimated marginal means for knowledge, attitude, and practice showed a non-significant result.

**Table 4:** Comparison of KAP score for RTI prevention among the intervention and control group based on time (Time-treatment interaction).

	**Comparison**	**Mean score**	**95% CI**	***P***
**Knowledge**				
Pre-intervention	Control	15.90	14.08, 17.77	0.885
	Intervention	15.73	13.92, 17.54	
Post-intervention	Control	16.38	14.94, 17.82	**0.005**
	Intervention	19.192	17.78, 20.61	
**Attitude**				
Pre-intervention	Control	31.70	30.24, 33.16	0.583
	Intervention	32.27	30.84, 33.70	
Post-intervention	Control	32.22	30.70, 33.74	**0.041**
	Intervention	34.44	32.95, 35.93	
**Practice**				
Pre-intervention	Control	26.10	24.53, 27.67	0.571
	Intervention	26.731	25.19, 28.27	
Post-intervention	Control	27.06	25.98, 28.27	**0.002**
	Intervention	29.46	28.40, 30.52	

*Repeated measures ANOVA between group analysis with regard to time was applied followed by pairwise comparison with 95% confidence interval adjustment by Bonferroni correction. Assumption of normality, homogeneity of variances, and compound symmetry were checked and were fulfilled*.

## Discussion

This study employed an interactive, self-operated, and novel smartphone health education module application to improve knowledge, attitude, and practice toward RTI prevention during Hajj among the participating pilgrims in the intervention group. The smartphone application was considered as the format of the intervention as the literature review provided several studies of successful digital-based interventions for health (31, 32). Striking instances include a digital health intervention, which has been reported to promote adherence to medical therapies and knowledge and understanding of cancer (33), and a smartphone application to enhance health knowledge, attitude, practice, and safety among University Students in Malaysia (34). The extensive choice of mobile phones highlights a vital chance to reshape health behaviors worldwide, especially in low- and middle-income nations (12). Conducting a study using a mobile phone technology to gather information on infections associated with mass gathering and travelers makes compliance with prevention practices achievable. It can also render useful data connected to health-related behavior (35).

The intervention program based on the generalizable modified HBM tested in this study showed significant benefits in terms of RTI prevention and increased health knowledge and health behavior. Furthermore, for participants who complied well with the health education module, the outcomes were significantly higher in the intervention group compared with the control subjects from baseline to post-intervention. These findings are also supported by a study conducted by Medley et al. (36). The intervention program based on the modified HBM employed in this study demonstrated remarkable achievement in terms of RTI prevention and advancement of health knowledge and health behavior. However, for pilgrims that participated and complied with the intervention module, the study outcomes were higher in the intervention arm when compared with the control arm from pre-intervention to the post-intervention. These results are also backed by findings from several studies (30, 37, 38).

There was an increase in the total knowledge scores for both intervention and control groups, which could be due to the general regular lectures and courses that are not guided by any health behavior theory given to pilgrims attending private Hajj company's regular orientation. Also, the billboards, signages, and posters displayed in Makkah and other Hajj areas. Similarly, Saudi authorities have made strict requirements and recommendations for visitors for Hajj to be in optimum health condition and physically fit (39). However, the mean KAP scores of the intervention were higher when compared with those of the control group. Consequently, the health education module is efficient in the advancement of knowledge, attitude, and practice related to RTI prevention among the respondents. The findings of our study are consistent with the finding of Alexandrino, Santos (40) which concluded that intervention has a positive effect on changing RTI infection knowledge in community settings. However, the present study indicates that the health education intervention and its compliance during Hajj led to improved knowledge, an attitudinal change regarding prevention and improved preventive practices as was seen in some studies (41–43).

This study employed Mixed design ANOVA which permit the estimation of the interaction of group effects, time effects as well as random and fixed effects related to repeated measure in the study. The importance of this research as an organized health education intervention program via smartphone application on change in lifestyle that is focused and particular for Hajj pilgrims concerning RTIs is that it can be recognized for adoption in Malaysia and even beyond. To a certain degree in this study, the group x time interaction effect of our treatment on the knowledge, attitude, and practice outcomes improved statistically significant by the intervention. Therefore, the detected difference in the outcomes can potentially be due to the intervention provided. These findings are similar to the findings of a study conducted among respondents of the general Hajj population which showed a significant increase in knowledge toward healthy behaviors among pilgrims using health educators (43). This smartphone application had a small effect on the enhancing the attitude of respondents regarding RTIs prevention. Efforts at enhancing attitude regarding RTIs will ultimately produce an assertive influence on RTIs prevention among Hajj pilgrims. Regarding the prevention practices, our smartphone application, the effect size was within partial eta square cut-off score for a moderate effect.

To the best of our knowledge, this is the first study to evaluate the effect of a smartphone application for health education intervention in improving knowledge, attitude, and practice regarding RTIs among Hajj pilgrims in Malaysia. This study suggests a strong biologically plausible and significant relationship between our intervention and study outcomes. The beneficial effect of intervention reported in this study has substantial implications for attaining the control and prevention of RTIs during mass gatherings. The data analysis employed in this study ensured the estimation of the combination of fixed effects, random effects, and repeated measure in the analysis. Several steps were ensured to reduce bias and contamination despite the lack of randomization and blinding. It had been hypothesized that the practice of RTI prevention by Hajj pilgrim operates through the HBM, and an intervention guided by this model would positively affect these constructs, leading to improvements in preventive practices.

However, pilgrims that participated in this study were all from Malay ethnic group from one state that is predominantly a Muslim state in Malaysia, as such they may not be fully representative of pilgrims from Malaysia. The interpretation of the results from this research should be accomplished with prudence due to intrinsic deficiencies and hindrances in regarding its implementation. These points raised could limit the generalizability of our conclusions to the pilgrims from Malaysia, despite the use of purposive sampling. The importance of this research as a structured health education intervention program on change in lifestyle that is focused and particular for Hajj pilgrims regarding RTIs is that it can be recognized for adoption in Malaysia and even beyond. Further studies are needed in other parts of Peninsular Malaysia, as well as in Eastern Malaysia.

## Conclusion

The health education module used in this study is considered as unique as it combines a panel of experts from a diverse group of specialists. Furthermore, the concept of its delivery and intervention is particularly unique by utilizing a new mobile phone application approach. Trained and skilled research assistants were recruited to deliver the module and ease of communication in Bahasa Malaysia spoken by most the targeted participants. The total knowledge, attitude, and practice scores were significantly high for the participants in the intervention arm when compared to those in the control arm. A such it can be inferred that the intervention was efficient in enhancing the outcomes of the study, though some improvement is still needed. The module developed is recommended to be included as a strategy and sensitization of participants during a mass gathering against RTIs.

## Data Availability Statement

The raw data supporting the conclusions of this article will be made available by the authors, without undue reservation.

## Ethics Statement

The studies involving human participants were reviewed and approved by Universiti Sains Malaysia, Ethics Committee for Research Involving Human Subjects (Ref no: USM/JEPeM/17020146). Universiti Sultan Zainal Abidin Malaysia human research ethics committees (UniSZA/UHREC/2019/88). The trial was registered with Australian New Zealand Clinical Trials Registry (ANZCTR) with registration number ACTRN12619000217101. The patients/participants provided their written informed consent to participate in this study.

## Author Contributions

MG, HH, and ZD conducted the survey and drafted the initial manuscript. NN, HH, NW-A, MA, and WA designed and supervised the study. AB and BA helped in the manuscript revision. All authors have read and approved the final manuscript.

## Conflict of Interest

The authors declare that the research was conducted in the absence of any commercial or financial relationships that could be construed as a potential conflict of interest.
